# Epicardial Dispersion of Repolarization Promotes the Onset of Reentry in Brugada Syndrome: A Numerical Simulation Study

**DOI:** 10.1007/s11538-023-01124-9

**Published:** 2023-02-15

**Authors:** Simone Scacchi, Piero Colli Franzone, Luca F. Pavarino, Vincenzo Gionti, Cesare Storti

**Affiliations:** 1 Dipartimento di Matematica, Università degli Studi di Milano, Via Saldini 50, 20133 Milan, Italy; 2 Dipartimento di Matematica, Università degli Studi di Pavia, Via Ferrata 1, 27100 Pavia, Italy; 3 Istituto di cura Città di Pavia, via Parco Vecchio 27, 27100 Pavia, Italy

**Keywords:** Brugada syndrome, Bidomain model, Reentrant ventricular arrhythmias, Parallel numerical simulations, GPU computing

## Abstract

The Brugada syndrome (BrS) is a cardiac arrhythmic disorder responsible for sudden cardiac death associated with the onset of ventricular arrhythmias, such as reentrant ventricular tachycardia and fibrillation. The mechanisms which lead to the onset of such electrical disorders in patients affected by BrS are not completely understood, yet. The aim of the present study is to investigate by means of numerical simulations the electrophysiological mechanisms at the basis of the morphology of electrocardiogram (ECG) and the onset of reentry associated with BrS. To this end, we consider the Bidomain equations coupled with the ten Tusscher–Panfilov membrane model, on an idealized wedge of human right ventricular tissue. The results have shown that: (1) epicardial dispersion of repolarization, generated by the coexistence of regions of early and late repolarization, due to different modulation of the $I_\mathrm{{CaL}}$ current, produces ECG waveforms exhibiting qualitatively the typical BrS morphology, characterized by ST elevation and partially negative T-waves; (2) epicardial dispersion of repolarization promotes the onset of reentry during the implementation of the programmed stimulation protocol, because of the conduction block occurring when a premature beat reaches the border of late repolarizing regions; and (3) the modulation of the $I_\mathrm{{to}}$ current affects the duration of reentry, which becomes sustained with a remarkable increase of $I_\mathrm{{to}}$ in the subepicardial layers.

**Supplementary Information:**

The online version contains supplementary material available at 10.1007/s11538-023-01124-9.

## Introduction

The Brugada syndrome (BrS), first described as a new clinical entity in Brugada and Brugada (1992), has attracted great interest because of its association with the risk of sudden cardiac death and susceptibility to ventricular arrhythmias (VA) in young patients; see Antzelevitch (2001b), Antzelevitch (2006), Benito et al. (2009) and Sieira et al. (2016). BrS is an inherited arrhythmogenic disease, transmitted as an autosomal dominant trait, and shows age- and sex-related penetrance. Clinical manifestations of the disease are more frequent in young adults, and they are eightfold more frequent in men than in women. At least 12 genes have been associated with BrS, but only two (SCN5A and CACN1Ac) individually account for more than 5$\%$ of positively genotyped patients. Diagnosis of BrS is based on a typical electrocardiographic pattern, characterized by an ST segment elevation of about 2 mm followed by a negative T-wave in the right precordial leads (V1–V3), observed either spontaneously or during a sodium channel blocker test (Fowler and Priori 2009; Priori et al. 2002). The incidence of arrhythmic events in patients with BrS was 13.5$\%$ per year in patients with a history of sudden cardiac arrest, 3.2$\%$ per year in patients with syncope and 1$\%$ per year in asymptomatic patients (Fauchier et al. 2013). The mechanisms underlying the characteristic electrocardiographic features and the onset of such arrhythmic events remain poorly understood. For this reason, it is difficult to determine whether a patient is at risk of undergoing arrhythmic events, i.e., to perform the so-called arrhythmic risk stratification. Accurate identification and treatment of individuals at high risk of sudden death are major challenges in the clinical management of BrS patients.

Recent studies in selected patients with Brugada syndrome have described complex arrhythmic substrates in the right ventricular outflow. The development of our work has been inspired by the research published by Nademanee et al. (2017) and Pappone et al. (2018), who explored clinical and electrophysiological predictors of malignant ventricular tachyarrhythmia inducibility in BrS. In particular, they investigated the correlations between the presence and extent of a region of cells with altered electrical properties (malignant VA substrate) in the right ventricle of BrS patients and the pathophysiological basis of lethal VA, which remain still unclear.

Because of the limitation to experimental research involving human cardiac tissue, alternative methods such as computer modeling are of great interest, see Bueno-Orovio et al. (2015), Hoogendijk et al. (2011), Tsumoto et al. (2020) and Xia et al. (2005). Previous computational studies, focusing on the numerical simulation of reentrant ventricular arrhythmias associated with BrS, have considered reduced tissue models, such as the isotropic Monodomain model (Bueno-Orovio et al. 2015), simplified geometries (Tsumoto et al. 2020; Caligari and Scacchi 2020) or phenomenological membrane models (Bueno-Orovio et al. 2015).

The aim of the present work is to investigate the electrophysiological mechanisms at the basis of the morphology of electrocardiogram (ECG) and the onset of reentry associated with BrS, by means of three-dimensional parallel numerical simulations. From the mathematical viewpoint, we have adopted: (1) the anisotropic Bidomain representation of the cardiac tissue, coupled with the human ten Tusscher membrane model; and (2) a parallel finite element solver with GPU acceleration.

The rest of the paper is organized as follows: Sects. 2 and 3 are devoted to the description of the mathematical models and numerical methods adopted for the numerical simulations; Sect. 4 presents the results of the numerical simulations, which are then discussed in Sect. 5.

## Mathematical Models

### The Bidomain Model

The macroscopic Bidomain representation of the cardiac tissue volume $\Omega$ is obtained by considering the superposition of two anisotropic continuous media, the intra- (i) and extra- (e) cellular media, coexisting at every point of the tissue and separated by a distributed continuous cellular membrane; see, e.g., Neu and Krassowska (1993), Pennacchio et al. (2006) for a derivation of the Bidomain model from homogenization of cellular models and Colli Franzone and Savaré (2002), Bourgault et al. (2009) and Veneroni (2009) for the well-posedness analysis. We recall that the cardiac tissue consists of an arrangement of fibers that rotate counterclockwise from epi- to endocardium, and that have a laminar organization modeled as a set of muscle sheets running radially from epi- to endocardium. The anisotropy of the intra- and extracellular media, related to the macroscopic arrangement of the cardiac myocytes in the fiber structure, is described by the anisotropic conductivity tensors $D_i(\textbf{x})$ and $D_e(\textbf{x})$, respectively, defined in (2).

We denote by $\Omega \subset \mathbb {R}^3$ the bounded physical region occupied by the cardiac tissue and introduce the parabolic–elliptic formulation of the Bidomain system. Given an applied intracellular current per unit volume $I_\mathrm{{app}}^i:\Omega \times (0,T)\rightarrow \mathbb {R}$, and initial conditions $v_0:\Omega \rightarrow \mathbb {R}$, $w_0:\Omega \rightarrow \mathbb {R}^{N_w}$, find the transmembrane potential $v:\Omega \times (0,T)\rightarrow \mathbb {R}$, extracellular potential $u_e:\Omega \times (0,T)\rightarrow \mathbb {R}$, the gating and ionic concentrations variables $w:\Omega \times (0,T)\rightarrow \mathbb {R}^{N_w}$ such that

1 $$\begin{aligned} \left\{ \begin{array}{ll} \chi \,c_m \displaystyle \frac{\partial v}{\partial t} - \mathrm{{div}}(D_i\nabla v) -\mathrm{{div}}(D_i\nabla u_e) +\chi \,I_\mathrm{{ion}}(v,w) = I_\mathrm{{app}}^i &{} \text{ in } \Omega \times (0,T)\\ -\mathrm{{div}}(D_i \nabla v)-\mathrm{{div}}((D_i+D_e) \nabla u_e) = 0 &{} \text{ in } \Omega \times (0,T)\\ \displaystyle \frac{\partial w}{\partial t} - R(v,w) = 0, &{} \text{ in } \Omega \times (0,T)\\ \textbf{n}^T D_i \nabla ( v+ u_e) = 0 &{} \text{ in } \partial \Omega \times (0,T)\\ \textbf{n}^T ( D_{i}+D_{e})\nabla u_{e}+ \textbf{n}^T D_{i} \nabla v = 0 , &{} \text{ in } \partial \Omega \times (0,T) \\ v(\textbf{x},0) = v_0(\textbf{x}), w(\textbf{x},0) = w_0(\textbf{x}) &{} \text{ in } \Omega , \end{array} \right. \end{aligned}$$

where $C_m$ is the membrane capacity, $\chi$ is the membrane surface to volume ratio and $\textbf{n}$ is the outward unit normal with respect to the domain boundary $\partial \Omega$. Since the extracellular potential $u_e$ is uniquely determined only up to a constant in space, we fix this constant by imposing the condition $\int _{\Omega } u_e \, \textrm{d}x = 0$. The nonlinear reaction term $I_\mathrm{{ion}}$ and the system of ordinary differential equations (ODEs) for the gating and ionic concentration variables *w* are given by the ionic membrane model; here, we will consider the ten Tusscher (TP06) membrane model (ten Tusscher et al. 2004; ten Tusscher and Panfilov 2006). $N_w$ denotes the number of gating and ionic concentration variables, which in the TP06 model amounts to 18.

The conductivity tensors $D_i(\textbf{x})$ and $D_e(\textbf{x})$ at any point $\textbf{x}\in \Omega$ are defined as

2 $$\begin{aligned} \begin{array} {ll} D_{i,e}(\textbf{x})&{} = \sigma ^{i,e}_l~ \textbf{a}_l(\textbf{x}) \textbf{a}_l^T(\textbf{x}) + \sigma ^{i,e}_t ~\textbf{a}_t(\textbf{x}) \textbf{a}_t^T(\textbf{x})+ \sigma ^{i,e}_n~ \textbf{a}_n(\textbf{x}) \textbf{a}_n^T(\textbf{x}) \\ &{} =\sigma _t^{i,e} I + \big (\sigma _l^{i,e} - \sigma _t^{i,e}\big ) \textbf{a}_l(\textbf{x}) \textbf{a}_l^T(\textbf{x}) + \big (\sigma _n^{i,e} - \sigma _t^{i,e}\big ) \textbf{a}_n(\textbf{x}) \textbf{a}_n^T(\textbf{x}). \end{array} \end{aligned}$$

Here $\textbf{a}_l(\textbf{x}), ~\textbf{a}_t(\textbf{x}),~\textbf{a}_n(\textbf{x})$, is a triplet of orthonormal principal axes with $\textbf{a}_l(\textbf{x})$ parallel to the local fiber direction, $\textbf{a}_t(\textbf{x})$ and $\textbf{a}_n(\textbf{x})$ tangent and orthogonal to the radial laminae, respectively, and both being transversal to the fiber axis (see, e.g., LeGrice et al. 1995). Moreover, $\sigma ^{i,e}_l,~\sigma ^{i,e}_t,~\sigma ^{i,e}_n~$ are the conductivity coefficients in the intra- and extracellular media measured along the corresponding directions $\textbf{a}_l, \textbf{a}_t, \textbf{a}_n$.

### Computation of Pseudo-electrocardiograms

In order to compute the extracardiac electrocardiograms (ECGs), we adopt the infinite medium approximation, which consists of assuming that both the bulk medium (intra- and extracellular) and extracardiac domains are isotropic media, with the same conductivity coefficient $\sigma _b$, and the extracardiac domain is unbounded; thus, $D_i+D_e = \sigma _b I$. As a consequence, with these simplifications, given a transmembrane potential distribution *v* inside the cardiac domain $\Omega$, the extracardiac/extracellular potential *u* in $\mathbb {R}^3$ satisfies the following differential problem:

3 $$\begin{aligned} -\sigma _b \Delta u(\textbf{x}) = \left\{ \begin{array}{ll} \mathrm{{div}}(D_i (\textbf{x}) \nabla v (\textbf{x})) &{} \quad \textbf{x}\in \Omega \quad \\ 0 &{} \quad \textbf{x}\in \mathbb {R}^3{\setminus }\Omega , \end{array} \right. \end{aligned}$$

where $\sigma _b$ is the conductivity of the extracardiac/bulk medium. Thus, by exploiting the fundamental solution of the three-dimensional Laplace equation in $\mathbb {R}^3$, the electric potential *u* at an extracardiac site $\textbf{x}$, called *pseudo-electrocardiogram* (pECG), is computed according to the following formula:

4 $$\begin{aligned} pECG(\textbf{x})=u(\textbf{x})=\frac{1}{4\pi \sigma _b}\int _\Omega D_i \nabla v \cdot \nabla \left( \frac{1}{|\textbf{x}-\textbf{y}|}\right) \mathrm{{d}}\textbf{y}. \end{aligned}$$

For further details, see Colli Franzone et al. (2014, Ch. 5, Proposition 5.1) and Geselowitz and Miller (1983).

### Variational Formulation

Let *V* be the Sobolev space $H^1(\Omega )$, define the spaces

$$ \widetilde{V}=\left\{ \psi \in V:\int _{\Omega }\psi =0\right\} \quad \text{ and } \quad U=V\times \widetilde{V}=\left\{ u=(\varphi ,\psi ):\varphi \in V, \psi \in \widetilde{V}\right\} , $$

define the usual $L^2$-inner product $(\varphi ,\psi )=\int _{\Omega }\varphi \psi d\textbf{x}\quad \forall \varphi ,\psi \in L^2(\Omega ),$ and the elliptic bilinear forms

$$\begin{aligned} a_{i,e}(\varphi ,\psi )= & {} \int _{\Omega }(\nabla \varphi )^T D_{i,e}(\textbf{x}) \nabla \psi \textrm{d}\textbf{x},\\ a(\varphi ,\psi )= & {} \int _{\Omega }(\nabla \varphi )^T D(\textbf{x}) \nabla \psi \textrm{d}\textbf{x}\quad \forall \varphi ,\psi \in H^1(\Omega ), \end{aligned}$$

where $D=D_i+D_e$ is the bulk conductivity tensor.

In order to simplify the notation, in the following we will denote $c_m=\chi \,C_m$ and $i_\mathrm{{ion}} = \chi \,I_\mathrm{{ion}}$. The variational formulation of the Bidomain model reads as follows. Given $v_0, w_0\in L^2(\Omega )$, $I_\mathrm{{app}}^i\in L^2(\Omega \times (0,T))$, find $v\in L^2(0,T;V)$, $u_e\in L^2(0,T;\widetilde{V})$ and $w\in L^2(0,T;L^2(\Omega )^{N_w})$ such that $\displaystyle \frac{\partial v}{\partial t}\in L^2(0,T;V)$, $\displaystyle \frac{\partial w}{\partial t}\in L^2(0,T;L^2(\Omega )^{N_w})$ and $\forall t\in (0,T)$

5 $$\begin{aligned} \left\{ \begin{array}{l} c_m \displaystyle \frac{\partial }{\partial t}(v,\hat{v}) + a_i(v,\hat{v}) + a_i(u_e,\hat{v})+(i_\mathrm{{ion}}(v,w),\hat{v}) = \big (I_\mathrm{{app}}^i,\hat{v}\big ) \quad \forall \hat{v}\in V\\ a_i(v,\hat{u}_e) + a(u_e,\hat{u}_e) = 0 \quad \forall \hat{u_e}\in \widetilde{V}\\ \displaystyle \frac{\partial }{\partial t}(w,\hat{w}) - (R(v,w),\hat{w}) = 0, \quad \forall \hat{w}\in V,\\ \end{array} \right. \end{aligned}$$

with the appropriate initial conditions as in (1).

## Numerical Methods

In this section, we briefly describe our numerical approximation of the Bidomain model; we refer the interested reader to Vigmond et al. (2002), Colli Franzone and Pavarino (2004), Colli Franzone et al. (2005, 2014, 2018) for further details.

### Space Discretization

System (5) is first discretized in space by the finite element method. Let $\mathcal {T}_h$ be a quasi-uniform triangulation of $\Omega$ having maximal diameter *h* and $V_h$ be an associated conforming finite element space. Once a finite element basis $\{\varphi _p\}_{p=1}^N$ of $V_h$ is chosen, denote by $M=\{m_{pj}\}$ the diagonal mass matrix obtained by the usual mass-lumping technique and by $A_{i,e}=\{a^{i,e}_{pj}\}$ the symmetric intra- and extracellular stiffness matrices, with elements

$$ a^{i,e}_{pj}=\int _{\Omega }D_{i,e}\nabla \varphi _j\cdot \nabla \varphi _p\ \textrm{d}x. $$

The semi-discrete Bidomain problem, obtained by applying a standard Galerkin procedure, can be written in compact matrix form as

6 $$\begin{aligned} \left\{ \begin{array}{l} \displaystyle c_m \mathcal {M}\frac{\textrm{d}}{\textrm{d}t} \left[ \begin{array}{c}\textbf{v}\\ \textbf{u}_e\\ \end{array}\right] +\mathcal {A} \left[ \begin{array}{c}\textbf{v}\\ \textbf{u}_e\\ \end{array}\right] +\left[ \begin{array}{c}M\textbf{I}_\mathrm{{ion}}(\textbf{v},\textbf{w})\\ \textbf{0}\\ \end{array}\right] = \left[ \begin{array}{c}M\textbf{I}^i_\mathrm{{app}}\\ \textbf{0}\\ \end{array}\right] \\ \\ \displaystyle \frac{\textrm{d}\textbf{w}}{\textrm{d}t}=\textbf{R}(\textbf{v},\textbf{w}),\\ \end{array} \right. \end{aligned}$$

with block mass and stiffness matrices

$$ \mathcal {M}=\left[ \begin{array}{ll}M &{}0\\ 0&{}0\\ \end{array}\right] ,\qquad \mathcal {A}=\left[ \begin{array}{cc}A_i &{}A_i\\ A_i&{}A_i+A_e\\ \end{array}\right] , $$

where $\textbf{v}$, $\textbf{u}_e$, $\textbf{w} = (\textbf{w}_1,\dots ,\textbf{w}_{N_w})^T$, $\textbf{R}(\textbf{v},\textbf{w})=(R_1(\textbf{v},\textbf{w}),\dots ,R_{N_w}(\textbf{v},\textbf{w}))^T$, $\textbf{I}_\mathrm{{ion}}(\textbf{v},\textbf{w})$ and $\textbf{I}^i_\mathrm{{app}}$ are the coefficient vectors of the finite element approximations of *v*, $u_e$, *w*, $R(v,w_1,...,w_{N_w})$, $i_\mathrm{{ion}}(v,w_1,...,w_{N_w})$ and $I_\mathrm{{app}}^i$, respectively.

### Time Discretization

As time discretization, we employ an implicit–explicit (IMEX) strategy, based on decoupling the ODEs from the PDEs and on treating the linear diffusion terms implicitly and the nonlinear reaction terms explicitly. The implicit treatment of the diffusion term is needed in order to avoid a stability constraint on the time step $\Delta t$ induced by the fine mesh size *h*. Nevertheless, due to the explicit treatment of the reaction terms, stability could be preserved for a time step $\Delta t$ satisfying a condition of Courant–Friedrichs–Lewy (CFL) type; see, e.g., Quarteroni and Valli (1994). The two equations in (6) arising from the discretization of the PDEs are solved uncoupled. In particular, at the general time step, given $\textbf{w}^n$, $\textbf{v}^n$ and $\textbf{u}_e^n$ computed at the previous time step,

- we first update the gating and ionic concentration variables $\textbf{w}^{n+1}$ by solving the ODEs of the membrane model,
- then we solve the elliptic equation computing $\textbf{u}_e^{n+1}$,
- and finally we update the transmembrane potential $\textbf{v}^{n+1}$ by solving the parabolic equation.

Summarizing in formulae, given $\textbf{w}^n,\ \textbf{v}^n, \textbf{u}_e^n$, the scheme is

$$ \begin{array}{lll} \displaystyle \textbf{w}^{n+1}+\Delta t \textbf{R}\big (\textbf{v}^n,\textbf{w}^{n+1}\big )&{}=&{}\textbf{w}^n\\ \displaystyle (A_i+A_e)\textbf{u}_e^n&{}=&{}\displaystyle -A_i\textbf{v}^n\\ \displaystyle \left( \frac{c_m}{\Delta t}M+A_i\right) \textbf{v}^{n+1}&{}=&{} \displaystyle \frac{c_m}{\Delta t}M\textbf{v}^n-A_i\textbf{u}_e^n+M\textbf{I}_\mathrm{{ion}}\big (\textbf{v}^n,\textbf{w}^{n+1}\big )+M\textbf{I}_\mathrm{{app}}^{i,n}. \end{array} $$

As a consequence, at each time step we solve once the linear system with matrix $A_i+A_e$ arising from the elliptic equation, and once the linear system with matrix $\frac{c_m}{\Delta t}M+A_i$ arising from the parabolic equation. Both linear systems are solved by the preconditioned conjugate gradient (PCG) method, since the matrices are symmetric positive definite in the parabolic case and positive semi-definite in the elliptic case. The preconditioner used for the parabolic system is Block Jacobi (BJ), because the related matrix is well conditioned, while for the elliptic system we use an algebraic multigrid preconditioner.

### Computational Domain, Discretization and Tissue Parameters

**Fig. 1:**
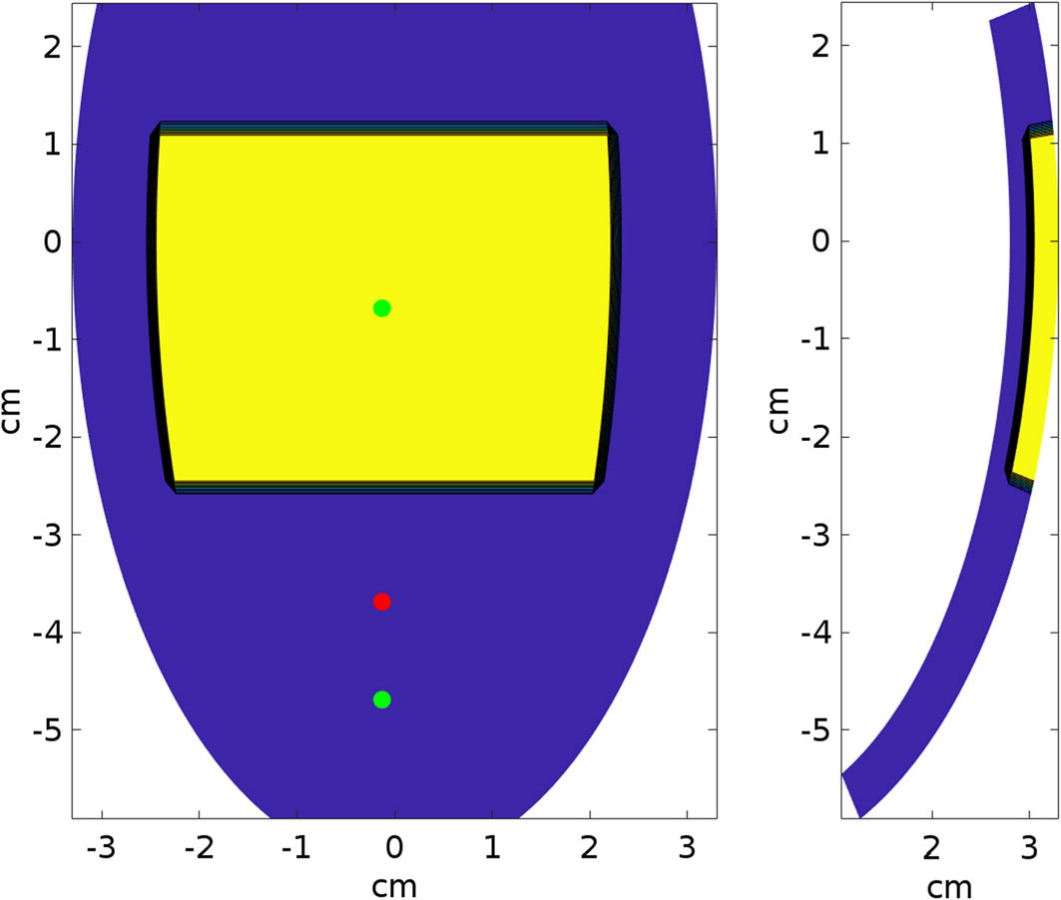
Left panel: epicardial view of the malignant VA substrate (yellow region), with stimulation site (red dot) and two exploring sites (green dots), where we have computed the transmembrane and action potential waveforms reported in Figs. 9 and 11. Right panel: transmural longitudinal view of the malignant VA substrate (yellow region) (Color figure online)

The right ventricular wedge domain $\Omega$ is the image of a Cartesian slab using ellipsoidal coordinates, described by the parametric equations

$$ \left\{ \begin{array} {ll} x = a(r)\cos \theta \cos \phi &{}\qquad \phi _\mathrm{{min}} \le \phi \le \phi _\mathrm{{max}}, \\ y = b(r)\cos \theta \sin \phi &{}\qquad \theta _\mathrm{{min}} \le \theta \le \theta _\mathrm{{max}}, \\ z = c(r)\sin \theta &{} \qquad 0\le r\le 1, \end{array} \right. $$

where $a(r) = a_1 + r ( a_2 - a_1 ),\ b(r) = b_1 + r ( b_2 - b_1),\ c(r) = c_1 + r ( c_2 - c_1 ),$ $a_1=b_1=2.8\ \textrm{cm},\, a_2=b_2=3.3\ \textrm{cm},\, c_1=5.9\ \textrm{cm},\ c_2=6.4\ \textrm{cm}$, and $\phi _\mathrm{{min}}=-\pi /2,\, \phi _\mathrm{{max}}=\pi /2, \theta _\mathrm{{min}}=-3\pi /8,\, \theta _\mathrm{{max}}=\pi /8$; see Fig. 1. We will refer to the inner surface of the truncated ellipsoid ($r=0$) as endocardium and to the outer surface ($r=1$) as epicardium. In all computations, a structured grid of $256 \times 256 \times 24$ hexahedral isoparametric $Q_1$ finite elements of size $h \approx 0.02\ \textrm{cm}$ is used in space, for a total amount of $1\,651\,225$ mesh nodes. Fibers rotate transmurally, linearly with the depth and counterclockwise from epicardium to endocardium, for a total amount of $90^{\circ }$. For the semi-implicit discretization in time, we use a constant time step size $\Delta t = 0.05$ ms. We also assume a transversely isotropic tissue with conductivity coefficients

7 $$\begin{aligned} \begin{aligned}&\sigma _l^e= 2,{} & {} \sigma _l^i= 3 \\&\sigma _t^e= 1.3514,{} & {} \sigma _t^i= 0.31525\\&\sigma _n^e = \sigma _t^e,{} & {} \sigma _n^i = \sigma _t^i, \end{aligned} \end{aligned}$$

all given in mS. The membrane capacity is set to $C_m = 1\ \upmu \textrm{F}$, while the membrane surface to volume ratio is set to $\chi = 10^3\ \textrm{cm}^{-1}.$

### BrS Parameter Setting

In the ECG waveforms, a coved ST segment elevation pattern followed by a negative T-wave is considered diagnostic of the Brugada syndrome; see, e.g., Sieira et al. (2016, Fig. 1). A reduced function of cardiac sodium channel plays an important role in the mechanism of BrS: The sodium channel blockers can provoke or augment the Brugada ECG pattern and mutations in SCN5A, the gene encoding the $\alpha$-subunit of the cardiac sodium channel, are identified in $\sim$20$\%$ of patients (Fowler and Priori 2009; Priori et al. 2002).

The disorder more frequently observed in mutations in SCN5A is a decrease of sodium current $I_\mathrm{{Na}}$ (Clancy and Rudy 2002), which leads to an imbalance between the positive inward and outward currents at the end of the transient repolarization phase of the cell action potential. This imbalance should occur with decreases of inward sodium $I_\mathrm{{Na}}$ and L-type calcium $I_\mathrm{{CaL}}$ currents or an increase of outward potassium $I_\mathrm{{to}}$ current, which leads to a development of a characteristic notch and the loss of action potential dome. Thus, one of the ways in which arrhythmias may occur in Brugada syndrome is through the formation of a heterogeneous substrate, in which a region exhibits abnormal action potentials. Such a substrate might be responsible for the onset of reentrant arrhythmias, as suggested in Pappone et al. (2018).

Following the approach of Hoogendijk et al. (2011), where ST segment elevation is connected to reductions of sodium channel ($G_\mathrm{{Na}}$) and L-type calcium channel ($G_\mathrm{{CaL}}$) maximal conductances and an increase of potassium channel ($G_\mathrm{{to}}$) maximal conductance, in our simulations, we modify in the TP06 model $G_\mathrm{{Na}}$, $G_\mathrm{{CaL}}$ and $G_\mathrm{{to}}$ in the region of BrS cells.

In the following, we will divide the transmural wall of the computational domain into two parts of equal depths, denoted by subendocardial and subepicardial regions, respectively. In order to model transmural heterogeneities of the action potential duration (APD), we scale the $I_\mathrm{{Ks}}$ current of a factor 0.7 and 1.4 in the subendocardial and subepicardial regions, respectively. Moreover, we will denote as malignant VA substrate a portion of tissue, located in the basal upper subepicardial region, occupying about $4.5\times 3.5\ \textrm{cm}^2$ of epicardial surface and 50% transmural depth, as displayed in Fig. 1.

We consider the following different settings in the TP06 model:

- **control**: all parameters in the TP06 model are set to their default values as given in the original paper (ten Tusscher and Panfilov 2006);
- **BRU1**: $G_\mathrm{{Na}}$ is set in the subepicardial region at 40$\%$ of its normal value;
- **BRU2**: in addition to the **BRU1** condition, $G_\mathrm{{CaL}}$ is set in the subepicardial region at 10$\%$ of its normal value;
- **BRU3**: in addition to the **BRU2** conditions, $G_\mathrm{{CaL}}$ is set in the malignant VA substrate at 120$\%$ of its normal value;
- **BRU4**: in addition to the **BRU3** conditions, $G_\mathrm{{to}}$ is set in the subepicardial region at 400$\%$ of its normal value.

**Fig. 2:**
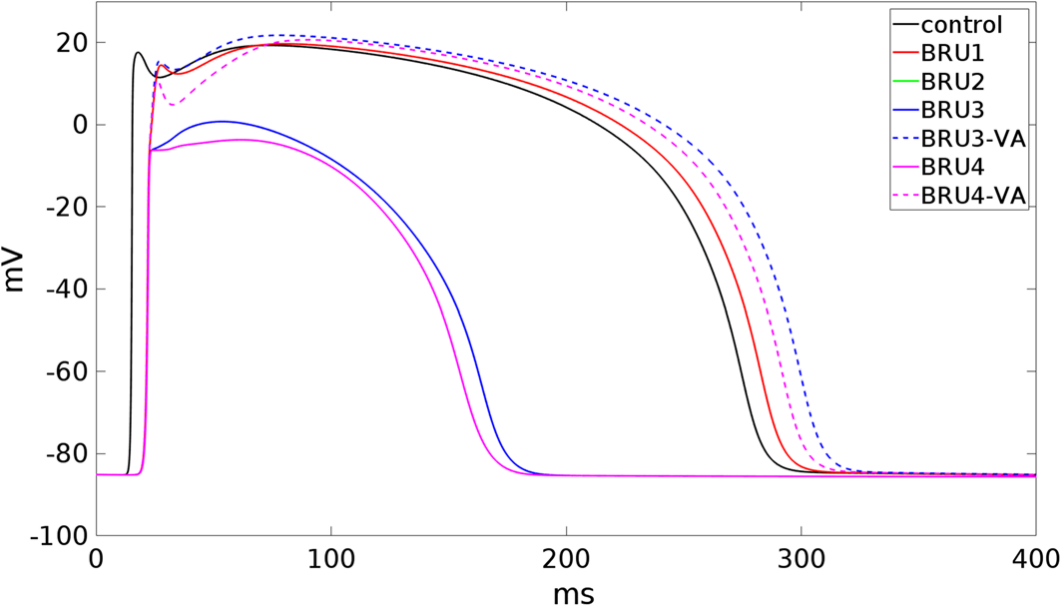
Epicardial action potential waveforms in the different BrS settings. For the **BRU3** and **BRU4** cases, we distinguish between sites located inside the malignant VA substrate (**BRU3**-VA and **BRU4**-VA) and outside (**BRU3** and **BRU4**). Note that the waveforms of the **BRU2** and **BRU3** coincide because the only difference between the two settings is the presence of the malignant VA substrate, thus outside it the cell membrane is the same

The corresponding subepicardial action potential waveforms, computed by solving the ODEs of the TP06 model applying the stimulus at a basic cycle length (BCL) of $500\ \textrm{ms}$, are reported in Fig. 2.

### Stimulation Protocol

Arrhythmic risk stratification in BrS is still challenging, especially in asymptomatic cases. In general, patients with documented ventricular fibrillation or with unexplained syncope should receive an implantable cardioverter defibrillator. However, for asymptomatic individuals with spontaneous type 1 BrS ECG, the best approach is still unclear. Pedro and Josep Brugada were the first to propose in 2002 (Brugada et al. 2003), on the basis of data from their registry, that arrhythmia inducibility at programmed electrical stimulation (PES) could be useful to identify patients at high risk.

For our simulations we have decided to use the same pacing procedure introduced in Brugada et al. (2003). Except where otherwise stated, the excitation process is started by applying a stimulus of 350 $\textrm{mA}/\textrm{cm}^3$ for 1 ms on a small area $0.12 \times 0.12 \times 0.06$ $\textrm{cm}^3$ at the epicardial site indicated in Fig. 1. For each simulation setting, we first apply four pacing stimuli (S1) at a BCL of $500\ \textrm{ms}$. Then, a premature stimulus (S2) is delivered $350\ \textrm{ms}$ after S1. If S2 does not generate a reentrant arrhythmia, the S1–S2 coupling interval is shortened in steps of $10\ \textrm{ms}$ until arrhythmia is induced or S2 fails to trigger excitation. If arrhythmia is not induced, an additional S3, and if necessary S4 stimulus, is delivered in the same manner as S2 (initially delivered $350\ \textrm{ms}$ after the previous stimulus, and then shortened until arrhythmia is induced or the stimulus fails to induce arrhythmia). The criterion adopted for successful arrhythmia induction is the onset of a reentrant excitation that remains sustained up to the end of simulation time, set to 4 s after the last stimulus. The total cost of a simulation where the reentrant excitation is sustained up to 4 s amounts to approximately 15 h.

**Table 1:** Parallel solver performance on MARCONI100 cluster

MARCONI100 cluster								
#cores	#gpus	$T_\mathrm{{ass}}$	$T_\mathrm{{memb}}$	$\textrm{it}_p$	$T_p$	$\textrm{it}_e$	$T_e$	$T_\mathrm{{tot}}$
8	–	16.58	1.31	5	1.13	7	7.92	1.05e+4
16	–	8.36	6.48e−1	5	5.50e−1	5	2.75	4.02e+3
32	–	4.26	3.30e−1	5	2.88e−1	4	1.35	2.01e+3
64	–	2.43	1.73e−1	5	1.55e−1	10	1.63	2.00e+3
1	1	54.02	3.20e−3	10	1.82e−2	93	7.99e−1	9.15e+2

$T_\mathrm{{ass}}$: CPU time to assemble the stiffness and mass matrices; $T_\mathrm{{memb}}$: membrane model solving time, average over time steps; $\textrm{it}_p$: CG iterations to solve the elliptic linear system, average over time steps; $T_p$: CPU time to solve the parabolic linear system, average over time steps; $\textrm{it}_e$: CG iterations to solve the elliptic linear system, average over time steps; $T_e$: CPU time to solve the elliptic linear system, average over time steps; $T_\mathrm{{tot}}$: CPU time for the whole simulation. All CPU times are given in seconds. The simulation time is 50 ms, for a total amount of 1000 time steps

**Table 2:** Parallel solver performance on DGX cluster

DGX cluster								
#cores	#gpus	$T_\mathrm{{ass}}$	$T_\mathrm{{memb}}$	$\textrm{it}_p$	$T_p$	$\textrm{it}_e$	$T_e$	$T_\mathrm{{tot}}$
8	–	1.28	1.38e−1	6	3.00e−1	8	5.55	6.04e+3
16	–	6.69e−1	6.97e−2	6	2.46e−1	4	2.40	2.76e+3
32	–	3.42e−1	3.57e−2	6	1.25e−1	4	1.16	1.34e+3
64	–	2.70e−1	2.15e−2	6	1.35e−1	9	1.95	2.12e+3
1	1	9.19	4.90e−3	12	1.24e−2	21	1.36e−1	1.72e+2
2	2	5.29	3.90e−3	12	1.56e−2	20	1.50e−1	1.85e+2
4	4	2.60	2.60e−3	12	1.53e−2	17	1.06e−1	1.34e+2
8	8	1.43	2.10e−3	12	2.12e−2	9	6.27e−2	9.43e+1

$T_\mathrm{{ass}}$: CPU time to assemble the stiffness and mass matrices; $T_\mathrm{{memb}}$: membrane model solving time, average over time steps; $\textrm{it}_p$: CG iterations to solve the elliptic linear system, average over time steps; $T_p$: CPU time to solve the parabolic linear system, average over time steps; $\textrm{it}_e$: CG iterations to solve the elliptic linear system, average over time steps; $T_e$: CPU time to solve the elliptic linear system, average over time steps; $T_\mathrm{{tot}}$: CPU time for the whole simulation. All CPU times are given in seconds. The simulation time is 50 ms, for a total amount of 1000 time steps

## Results

We report in this section the results of parallel numerical simulations on the three-dimensional wedge of right ventricular tissue. Except where otherwise stated, the simulations have been performed on the Marconi100 and DGX Linux clusters at the CINECA laboratory (see below). Our *in-house* code is written in C and is based on the parallel libraries MPI and PETSc (Balay et al. 2016), developed at the Argonne National Laboratory (USA). A CUDA kernel has been developed and included in the C code to solve the ODEs of the membrane model on the GPUs.

The rest of the section is organized as follows: in Test 1, we study the performance of the parallel solver; in Test 2, we compute the activation, repolarization and APD spatial distribution in the five settings; in Test 3, we study how the different BrS parameter calibrations affect the pECG waveforms; in Test 4, we simulate the induction of reentry.

### Test 1: Performance of the GPU and CPU Bidomain Solvers

We compare the performance of the GPU Bidomain solver with our previous CPU solver. Two computational platforms have been considered:

- Marconi100, a Linux cluster at the Cineca laboratory, constituted by 980 nodes, each carrying $2\times 16$ cores IBM POWER9 AC922 at 3.1 GHz, $4 \times$ NVIDIA Volta V100 GPUs, Nvlink 2.0, 16GB per GPU, 256 GB RAM per node (https://www.hpc.cineca.it/hardware/marconi100);
- DGX, a Linux cluster at the Cineca laboratory, constituted by 3 nodes, each carrying $2\times 64$ AMD EPYC 7742 cores at 2.25 GHz 2 HTs, $8 \times$ NVIDIA A100 Tensor Core GPUs, Nvlink 3.0, 40GB per GPU, 1 TB RAM per node (https://www.hpc.cineca.it/hardware/dgx).

In the Marconi100 cluster the GPU computations have been performed using only one core and one GPU, while in the DGX cluster we have used up to eight cores and eight GPUs. On both clusters, the computations have been executed on a single node. In the GPU solver, the parabolic system is preconditioned by the Jacobi preconditioner, while the elliptic system is preconditioned by the PCGAMG algebraic multigrid preconditioner provided by the PETSc library (Balay et al. 2016). In the CPU solver, the parabolic system is preconditioned by the Block Jacobi preconditioner, while the elliptic system is preconditioned by the BoomerAMG algebraic multigrid preconditioner (Henson and Yang 2002) provided by the HYPRE library (https://www.llnl.gov/CASC/hypre/). The simulation time is 50 ms, for a total amount of 1000 time steps.

The results obtained on Marconi100, reported in Table 1, show that: the GPU membrane solver is about 50 times faster than the 64 cores CPU membrane solver; the GPU parabolic solver is about 8 times faster than the 64 cores CPU parabolic solver; the GPU elliptic solver is about twice as fast as the 64 cores CPU elliptic solver; as a result, the GPU Bidomain solver is in total about twice as fast as the 64 cores CPU elliptic solver.

The results obtained on DGX, reported in Table 2, show that: the GPU membrane solver is about 10 times faster than the 64 cores CPU membrane solver; the GPU parabolic solver is about 6 times faster than the 64 cores CPU parabolic solver; the GPU elliptic solver is about 31 times faster than the 64 cores CPU elliptic solver; as a result, the GPU Bidomain solver is in total about 22 times faster than the 64 cores CPU elliptic solver.

**Fig. 3:**
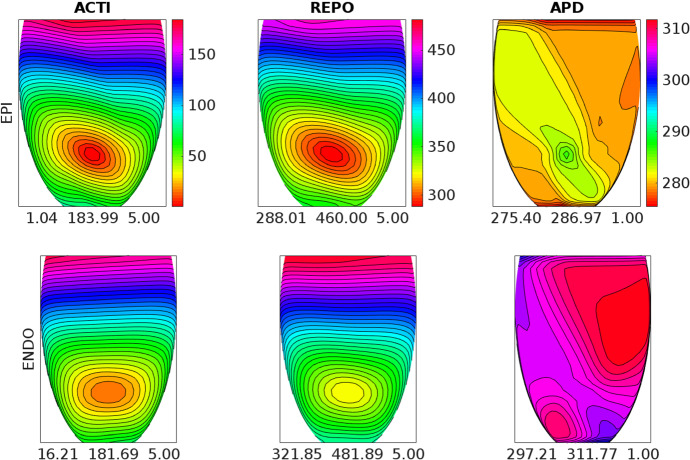
Activation time (ACTI), repolarization time (REPO) and action potential duration (APD) distributions computed after an endocardial stimulation in the control setting. Each ENDO panel has the same colorbar of the associated EPI panel above. The three numbers reported below each panel are the minimum, maximum and step in ms of the displayed map

**Fig. 4:**
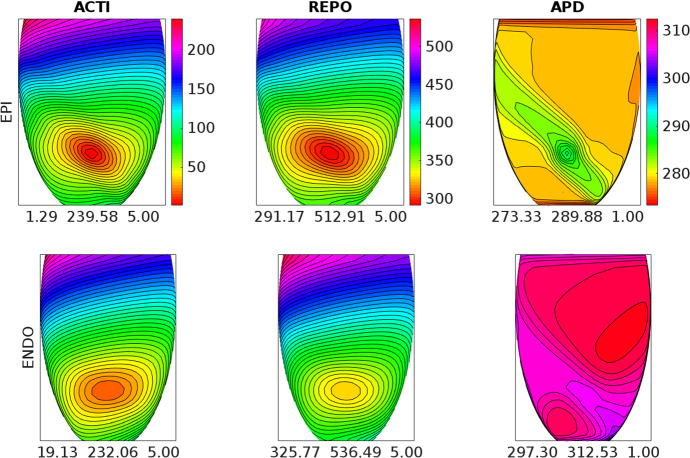
Activation time (ACTI), repolarization time (REPO) and action potential duration (APD) distributions computed after an endocardial stimulation in the **BRU1** setting. Same format as in Fig. 3

**Fig. 5:**
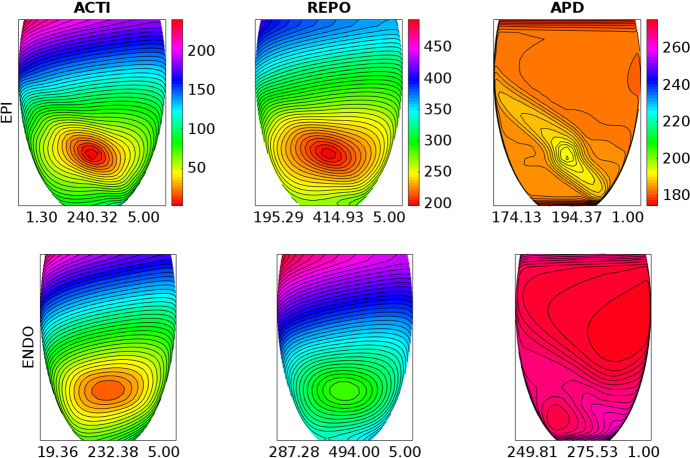
Activation time (ACTI), repolarization time (REPO) and action potential duration (APD) distributions computed after an endocardial stimulation in the **BRU2** setting. Same format as in Fig. 3

**Fig. 6:**
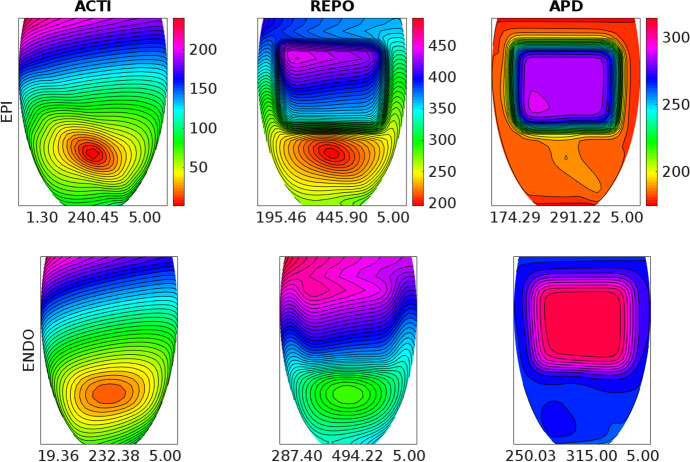
Activation time (ACTI), repolarization time (REPO) and action potential duration (APD) distributions computed after an endocardial stimulation in the **BRU3** setting. Same format as in Fig. 3

**Fig. 7:**
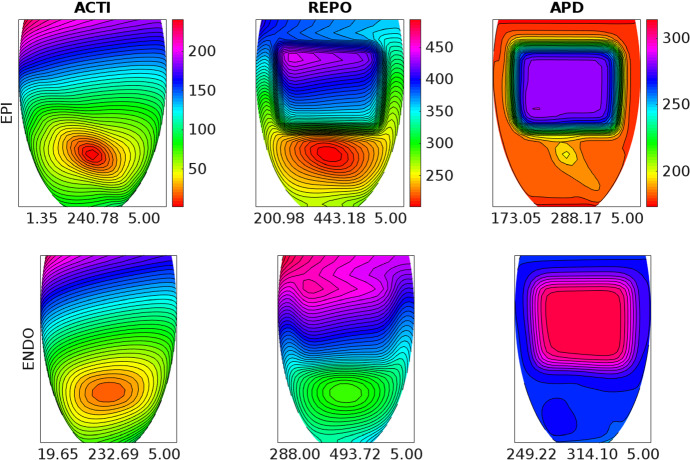
Activation time (ACTI), repolarization time (REPO) and action potential duration (APD) distributions computed after an endocardial stimulation in the **BRU4** setting. Same format as in Fig. 3

**Table 3:** Endocardial, epicardial and total dispersions of activation time, repolarization time and APD

Setting	$\textrm{dAT}_\mathrm{{endo}}$	$\textrm{dAT}_\mathrm{{epi}}$	$\textrm{dAT}_\mathrm{{tot}}$	$\textrm{dRT}_\mathrm{{endo}}$	$\textrm{dRT}_\mathrm{{epi}}$	$\textrm{dRT}_\mathrm{{tot}}$	$\textrm{dAPD}_\mathrm{{endo}}$	$\textrm{dAPD}_\mathrm{{epi}}$	$\textrm{dAPD}_\mathrm{{tot}}$
Control	165.5	183.0	183.0	160.0	172.0	193.9	14.6	11.6	36.4
BRU1	212.9	238.3	238.3	210.7	221.7	245.3	15.2	16.6	39.2
BRU2	213.0	239.0	239.0	206.7	219.6	298.7	25.7	20.2	101.4
BRU3	213.0	239.1	239.1	206.8	250.4	298.8	65.0	116.9	140.7
BRU4	213.0	239.4	239.4	205.7	242.2	292.7	64.9	115.1	141.1

All quantities are reported in ms

### Test 2: Activation and Repolarization Sequences and APD Distributions

The aim of this test is to study how the different BrS settings affect the activation and repolarization sequences and the APD distributions, after a unipolar epicardial stimulus applied at the location indicated in Fig. 1. Figures 3, 4, 5, 6 and 7 report the endocardial and epicardial activation time, repolarization time and APD distributions in the **control**, **BRU1**, **BRU2**, **BRU3** and **BRU4** settings, respectively. Table 3 reports the dispersion (maximum minus minimum) of the activation and repolarization time, and APD, computed on endocardium, epicardium and on the entire tissue.

We first observe that, in the **BRU1** setting, the activation sequence is about $57\ \textrm{ms}$ longer than in the **control** setting ($239.58\ \textrm{ms}$ (**BRU1**) vs. $183.99\ \textrm{ms}$ (**control**)). This delay is due to the reduction of the maximal conductance of the $I_\mathrm{{Na}}$ current. The slower activation induces a slower and longer repolarization sequence, that yields, especially on the epicardium, an increase of the APD dispersion of $5\ \textrm{ms}$ ($16.6\ \textrm{ms}$ (**BRU1**) vs. $11.6\ \textrm{ms}$ (**control**)).

In the **BRU2** setting, the activation sequence is comparable with that obtained in the **BRU1** setting. Due to the reduction of the maximal conductance of the $I_\mathrm{{CaL}}$ current in the subepicardial region, the epicardial APD is about 100 ms shorter than in the **BRU1** setting. As a consequence, the epicardial repolarization occurs much earlier than in the **BRU1** setting. The electrotonic effect induces a reduction of the repolarization time of about 40 ms on the endocardium, too. However, the endocardial and epicardial dispersions of repolarization remain comparable with those obtained in the **BRU1** setting, while the total dispersion of repolarization increases of about 3 ms (298.7 ms (**BRU2**) vs. 245.5 ms (**BRU1**)).

In the **BRU3** setting, the activation sequence is again comparable with the previous **BRU1** and **BRU2** settings. The presence of the subepicardial malignant VA substrate, with a larger $I_\mathrm{{CaL}}$ current than in the rest of the subepicardial region, yields an increase of the maximal epicardial APD of almost 100 ms with respect to the **BRU2** setting (291.22 ms (**BRU3**) vs. 194.37 ms (**BRU2**)). As a consequence, the epicardial dispersion of repolarization increases from 219.6 (**BRU2**) to 250.4 ms (**BRU3**). Sharp repolarization gradients appear at the boundaries of the malignant VA substrate. The electrotonic effect induces also a modification of the endocardial repolarization sequence, which presents concave isochrones corresponding to the endocardial projection of the malignant VA substrate. Nevertheless, the endocardial and total dispersions of repolarization remain comparable with those obtained in the **BRU2** setting.

Finally, the **BRU4** setting presents activation, repolarization and APD distribution and dispersions analogous to those of the **BRU3** setting.

**Fig. 8:**
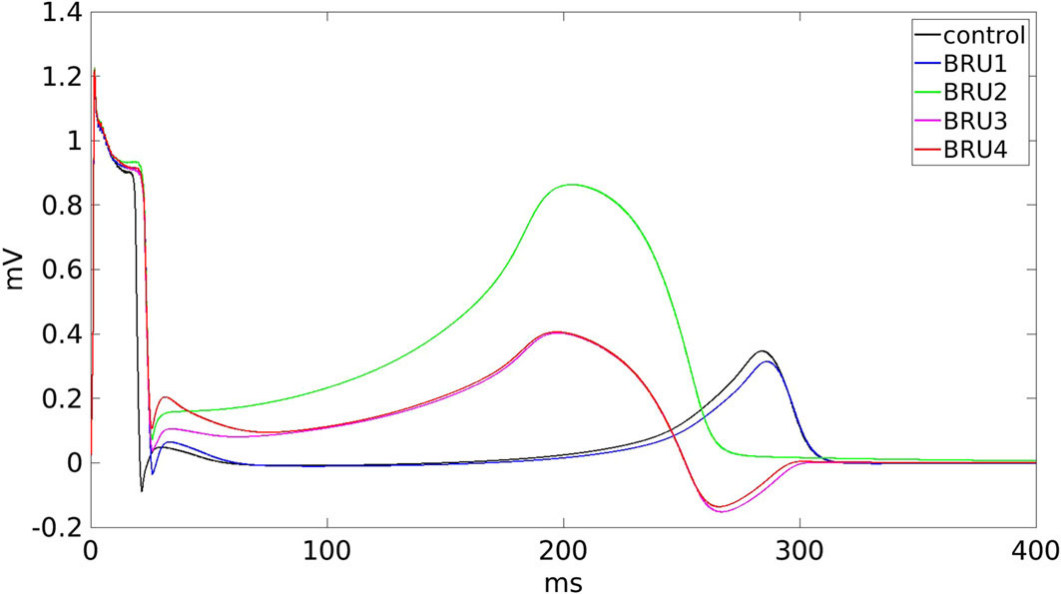
Pseudo-ECG waveforms computed at a distance of 2 cm from the center of the epicardial surface

### Test 3: Effects of BrS Model Calibration on the pECG

The aim of this test is to study the role played by the different BrS parameter settings **BRU1-4** on the genesis of the ECG morphology. To this end, we compute the pECG according to Eq. (4) in an extracardiac site $\textbf{x}$ located at a distance of 2 cm from the center of the epicardial surface. In these simulations, we mimic the sinus rhythm excitation by stimulating the entire endocardial surface.

In the **control** setting, the pECG waveform displayed in Fig. 8 exhibits the typical positive QRS complex, since the excitation wavefront propagates from endocardium toward epicardium. Then, after a flat ST interval, the T-wave presents a positive dome, since the subepicardial layers recover before the endocardial ones, due to the transmural APD heterogeneities.

In the **BRU1** setting, the pECG waveform is comparable with that computed in the **control** setting, except a longer QRS complex, due to the slower activation induced by the reduction of $I_\mathrm{{Na}}$ current.

In the **BRU2** setting, the pECG waveform exhibits a large ST elevation and a huge positive T-wave, because of the fast repolarization occurring in the subepicardial layers, where the $I_\mathrm{{CaL}}$ current is reduced.

In the **BRU3** setting, we observe again a marked ST elevation and an initially positive T-wave, due to the fast repolarization of the apical and central subepicardial layers. However, after about 250 ms, the pECG undergoes an inversion of the T-wave, which becomes negative, because of the late repolarization of the malignant VA substrate. Thus, in this setting, the pECG waveform exhibits the typical characteristics of a BrS ECG, with ST elevation and T-wave inversion.

Finally, in the **BRU4** setting, the pECG waveform is analogous to that computed in the **BRU3** setting, except a more prominent J-wave. Thus, this result suggests that the increase of $I_\mathrm{{to}}$ current in the subepicardial layers seems responsible for the presence of prominent J-waves in BrS ECGs.

**Fig. 9:**
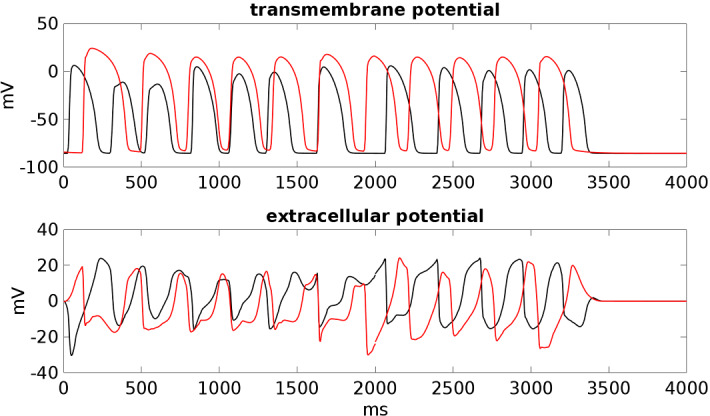
Transmembrane and extracellular potential waveforms computed from **BRU3** setting after S1–S2 stimulations, in two epicardial sites: one located inside the malignant VA substrate (red line) and one outside (black line), see Fig. 1. $t=0$ is the onset of the S1 stimulus. The S2 stimulus is applied at $t=260$ ms (Color figure online)

**Fig. 10:**
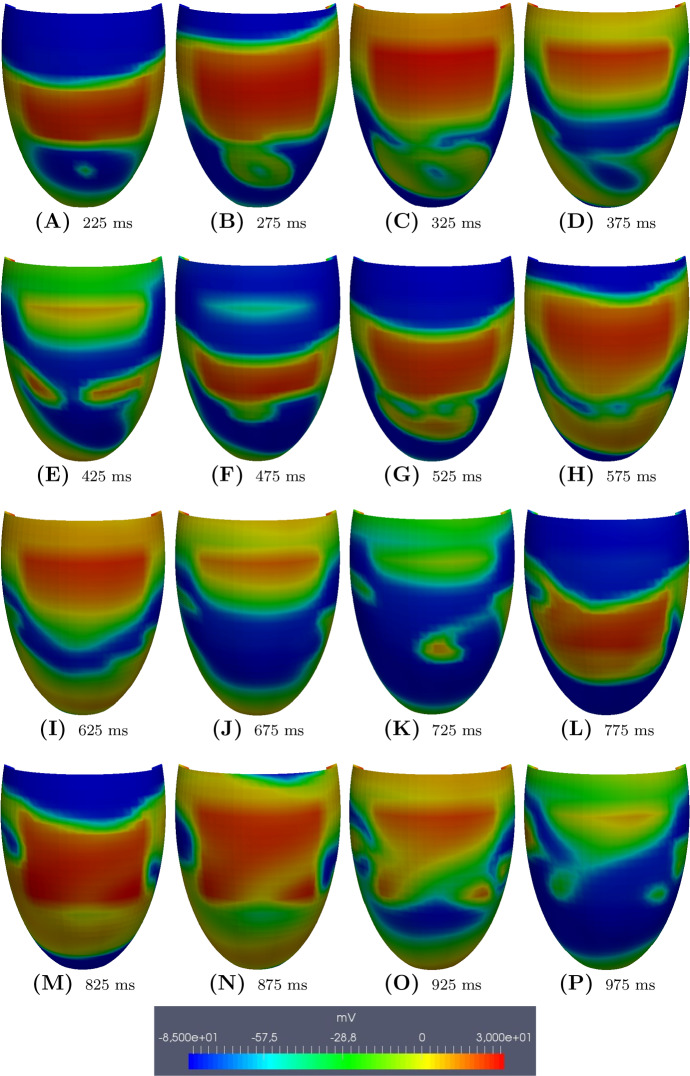
**BRU4** setting. Transmembrane potential snapshots (*t* = 225–975 ms) on the epicardial surface. $t = 0$ corresponds to the S2 stimulus. The S3 stimulus is applied at $t = 220$ ms

**Fig. 11:**
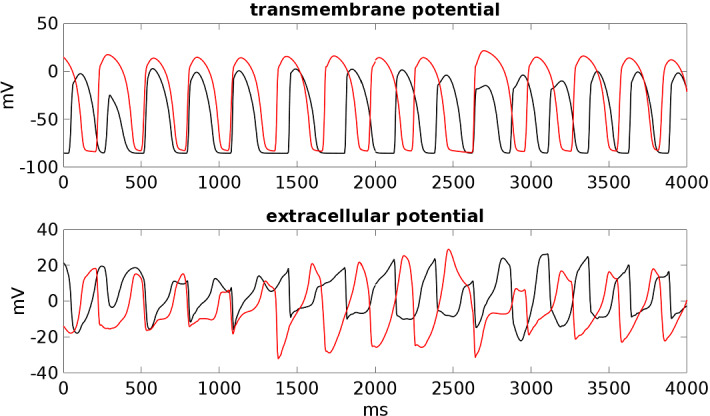
Transmembrane and extracellular potential waveforms computed from **BRU4** setting after S2–S3 stimulations, in two epicardial sites: one located inside the malignant VA substrate (red line) and one outside (black line), see Fig. 1. $t=0$ is the onset of the S2 stimulus. The S3 stimulus is applied at $t=220$ ms (Color figure online)

### Test 4: Induction of Reentry

In this test, we apply the PES protocol with a unipolar stimulating electrode at the epicardial site indicated in Fig. 1. In the **control**, **BRU1** and **BRU2** settings, we were not able to induce reentry.

In the **BRU3** setting, we applied an S2 stimulus at 260 ms after the S1 stimulus. See movie **SM_BRU3** in the Supplementary material. The excitation wavefront elicited from the S2 stimulus propagates through the epicardial surface until it reaches the bottom border of the malignant VA substrate, where a block of excitation occurs. The electric impulse then propagates around the malignant VA substrate, still refractory. When the tissue becomes excitable again, the wavefront enters the malignant VA substrate, generating a reentrant activation, which propagates backward toward the region of the excitation block and reexcites the epicardial tissue. The reentry is maintained for about 3 s, then it dies; see Fig. 9.

In the **BRU4** setting, we applied an S2 stimulus at 260 ms after the S1 stimulus and an S3 stimulus at 220 ms after the S2 stimulus. See Fig. 10 and movie **SM_BRU4** in the Supplementary material, starting from the S2 stimulus. The excitation wavefront elicited from the S3 stimulus propagates through the epicardial surface until it reaches the bottom border of the malignant VA substrate, where it is blocked because the tissue is still refractory. When the tissue becomes excitable again, the wavefront enters the malignant VA substrate, generating reentry. The reentry in this case is maintained until the end of the simulation at 4 s; see Fig. 11.

## Discussion

By means of parallel three-dimensional simulations, we have studied the electrophysiological mechanisms determining the ECG morphology and the onset of reentry associated with BrS. The cardiac domain considered is a three-dimensional wedge of the right ventricular wall. Despite the simplified geometry, we have taken into account the main features of cardiac electrophysiological modeling, i.e., the anisotropic Bidomain representation of the ventricular tissue, transmural fiber rotation, a mechanistic human ventricular membrane model, and transmural action potential heterogeneities. We have employed the Bidomain model instead of the Monodomain model in order to compute the electrograms inside the cardiac tissue and to have a more accurate representation of the cardiac sources, that might influence significantly the dynamics of reentry in pathological conditions. In order to induce reentry, we have implemented *in silico* the programmed electrical stimulation protocol, usually adopted in the clinical practice.

The results have shown that:

- Epicardial dispersion of repolarization, generated by the coexistence of regions of early and late repolarization (in the malignant VA substrate), due to different modulation of the $I_\mathrm{{CaL}}$ current, produces pECG waveforms exhibiting qualitatively the typical BrS morphology, characterized by ST elevation and partially negative T-waves;
- Epicardial dispersion of repolarization promotes the onset of reentry during the implementation of the PES protocol, because of the conduction block occurring when a premature beat reaches the border of the late recovering malignant VA substrate;
- The 400% increase of $I_\mathrm{{to}}$ in the subepicardial layers influences the morphology of the pECG, yielding a prominent J-wave, and the duration of reentry, which becomes sustained.

The presence of subepicardial regions of early repolarization, due to a severe reduction of $I_\mathrm{{CaL}}$ current, plays a decisive role in the genesis of ST elevation in the BrS ECG waveform. On the other hand, the concurrent presence of a sufficiently large subepicardial region of late repolarization induces the inversion of T-wave polarity, typical of BrS ECG waveforms, and promotes conduction blocks after premature beats, leading to the onset of reentry. This is in agreement with the experimental investigations (Nademanee et al. 2017; Pappone et al. 2018) and a previous computational study (Bueno-Orovio et al. 2015), where a phenomenological membrane model is adopted, together with a reduced isotropic Monodomain model.

The presence of prominent J-waves in some ECG leads, a characteristic of J-wave syndromes, i.e., a family of syndromes BrS belongs to, has been identified as a marker for a substrate capable of generating life-threatening ventricular arrhythmias, see, e.g., Antzelevitch and Yan (2010, 2015). Indeed, our computational results are in agreement with these experimental findings, because we have shown that large $I_\mathrm{{to}}$ values yield prominent J-waves and sustained reentry.

### Clinical Implications

The results of this study confirm the rationale of selective pharmacological modulation of $I_\mathrm{{to}}$ and $I_\mathrm{{CaL}}$ currents and/or of transcatheter ablation of the right ventricular epicardial substrate to reduce the likelihood of ventricular arrhythmias of BrS patients.

### Limitations

A simplified geometric wedge model of the right ventricular tissue has been employed in this study. The extension to a biventricular geometry, with the inclusion of a patient-specific characterization of the malignant substrate based on electro-anatomical mapping, would strengthen the results obtained in the present study. A further limitation of the study is the computation of the pECG instead of coupling the Bidomain equations with the Laplace equation in the surrounding medium. The rationale for this choice was the need of reducing the computational costs.

## Supplementary Information

Below is the link to the electronic supplementary material.

Supplementary file 1 (docx 5 KB)
