# Supplementary material for: Epicardial Dispersion of Repolarization Promotes the Onset of Reentry in Brugada Syndrome: A Numerical Simulation Study
Source: Bull Math Biol. 2023 Feb 15;85(3):22. doi: 10.1007/s11538-023-01124-9 (PMC9931802; doi:10.1007/s11538-023-01124-9)
Supplement: Supplementary file 1 — (docx 5 KB) [file 11538_2023_1124_MOESM1_ESM.docx]

**Supplementary material**

**Captions of the movies**

- **SM_BRU3** movie**: BRU3** setting, evolution of the epicardial transmembrane potential distribution after the S1 stimulus (t = 0 ms) and the S2 stimulus (t = 260 ms). The excitation wavefront elicited from the S2 stimulus propagates through the epicardial surface until it reaches the bottom border of the malignant VA substrate, where a block of excitation occurs. The electric impulse then propagates around the malignant VA substrate, still refractory. When the tissue becomes excitable again, the wavefront enters the malignant VA substrate, generating a reentrant activation, which propagates backward towards the region of the excitation block and re-excites the epicardial tissue. The reentry is maintained for about 3 s, then it dies.
- **SM_BRU4** movie**: BRU4** setting, evolution of the epicardial transmembrane potential distribution after the S2 stimulus (t = 0 ms) and the S3 stimulus (t = 220 ms). The excitation wavefront elicited from the S3 stimulus propagates through the epicardial surface until it reaches the bottom border of the malignant VA substrate, where it is blocked because the tissue is still refractory. When the tissue becomes excitable again, the wavefront enters the malignant VA substrate, generating reentry. The reentry in this case is maintained until the end of the simulation at 4 s.
